# Implementing health promotion interventions in a pediatric oncology setting: A qualitative study among families impacted by cancer and healthcare professionals

**DOI:** 10.1177/13674935251341008

**Published:** 2025-05-08

**Authors:** Catherine Demers, Isabelle Gélinas, Johanne Kerba, Keven Lee, Claude Julie Bourque, Kristopher Lamore, Isabelle Bouchard, Caroline Meloche, Caroline Laverdière, Daniel Curnier, Valérie Marcil, Serge Sultan, Daniel Sinnett, Johanne Higgins

**Affiliations:** 1 School of Physical and Occupational Therapy, 5620 McGill University, Montreal, QC, Canada; 2 70443 CHU Ste-Justine, Montreal, QC, Canada; 3 Occupational Therapy Department, Université du Québec à Trois-Rivières, Trois-Rivieres, QC, Canada; 4 Montreal Centre for Interdisciplinary Research in Rehabilitation (CRIR), Montreal, QC, Canada; 5 Department of Nutrition, Faculty of Medicine, 5622 Université de Montréal, Montreal, QC, Canada; 6 Department of Pediatrics, Faculty of Medicine, 5622 Université de Montréal, Montreal, QC, Canada; 7 Laboratoire de Psychopathologie et Processus de Santé, 26907 Université de Paris Cité, Paris, France; 8 CNRS, UMR 9193 – SCALab – Sciences Cognitives et Affectives, Université de Lille, Lille, France; 9 School of Kinesiology and Exercise Science, Faculty of Medicine, 5622 Université de Montréal, Montreal, QC, Canada; 10 Department of Psychology, 5622 Université de Montréal, Montreal, QC, Canada; 11 School of Rehabilitation, Faculty of Medicine, 5622 Université de Montréal, Montreal, QC, Canada

**Keywords:** health promotion', qualitative research’, cancer care facilities’, pediatrics'

## Abstract

One way to prevent or minimize adverse effects from pediatric cancer is to adopt and maintain healthy behaviours. However, there is limited knowledge on how to successfully implement health promotion (HP) interventions in clinical settings. This study aimed to determine the factors affecting the participation in and implementation of an HP program from the perspective of adolescents impacted by cancer, parents of children or adolescents impacted by cancer, and healthcare professionals (HCPs). We conducted semi-structured interviews with adolescents and parents, and focus groups with HCPs. Data were analysed using thematic analysis. A total of five adolescents, nine parents, and eleven HCPs were interviewed. Three major themes were determined: (1) facilitators to participation and implementation, (2) barriers to participation and implementation, and (3) suggestions for improvement. Factors identified as keys to participation include tailoring interventions to families’ specific needs and social support. Organizational barriers, health issues, and a lack of interest or need hampered participation in the program. Implementation was positively impacted by the interventions’ perceived relevance and negatively by their lack of integration in clinical care. While HP interventions hold promise for improving quality of life, successful implementation requires addressing the multifaceted challenges faced by participants and providers.

## Background

Following the increase in survival rate in pediatric oncology in the last decades, there has been a paradigm shift from attaining survival at all costs to survival with reduced acute, chronic, and late-onset toxicities (Williams et al., 2021). There is mounting evidence that children impacted by cancer are at a greater risk of suffering from various adverse effects secondary to cancer and its treatment (Erdmann et al., 2021; Galligan, 2017; Landier et al., 2015; Lemay et al., 2019). In addition, the current state of literature indicates that childhood cancer and its treatment have an important negative impact on the health behaviours of cancer survivors that also persist through time into survivorship (Nghiem et al., 2024). Such adverse repercussions not only have physical impacts such as obesity and metabolic disease (Barnea et al., 2015) but can also lead to mental health challenges such as anxiety, depression, and experiences of loneliness (Nathan et al., 2018; Papini et al., 2023). Targeting sustainable health behaviour changes thus represents a promising avenue for the prevention and mitigation of negative lasting impact of cancer. Research has shown that health promotion (HP) interventions positively impact health behaviours (Caru et al., 2020a; Wurz et al., 2021b) as well as some acute and long-term adverse effects (Hudson and Patte, 2008). Researchers and clinicians have emphasized the need for innovative HP interventions to lessen the impact of adverse effects (Beaulieu-Gagnon et al., 2019b; Caru et al., 2020b; Robison and Hudson, 2014) and for support for children impacted by cancer throughout their journey (i.e. from diagnosis to survivorship). In recent years, an increasing number of HP interventions have been developed in pediatric oncology, and reviews underscore their potential in modifying behaviours and improving health outcomes (Braam et al., 2016; Demers et al., 2021; Morales et al., 2018).

Research to support the integration of these interventions into pediatric oncology standard care is scarce (Phillips et al., 2022). Among these studies, most have focused on evaluating the interventions, overlooking implementation in real-life settings and sustainability following the study period (Daeggelmann et al., 2019). While efficacy research is important, there is an urgent need for research that focuses on how to best implement HP strategies in pediatric oncology clinical settings. For example, studies focusing on the views of children impacted cancer and survivors underscore these health initiatives should be personalized (Cai et al., 2024) and based on their needs and desires (Einberg et al., 2016). Identifying implementation challenges is important to understand why interventions fail or succeed, facilitating translation into real-world settings. Studies aiming to explore families’ experiences have contributed to understand how outcomes are achieved, which is crucial to maximizing the impact of intervention or services received (Heath et al., 2024; Long et al., 2010). It is important to address this dearth of knowledge on effective ways to promote healthy behaviours and on how families experience HP interventions while going through the pediatric cancer journey (Demers et al., 2021). Implementation research that focuses attention on identifying factors that influence adoption of healthy behaviours of families impacted by cancer is essential to better tailor current practices to the needs of those affected by cancer to effectively improve their health and well-being (Arroyave et al., 2008) and support interventions’ sustainability (Wurz et al., 2021a).

### Aim

The aim was to determine the factors affecting participation in an HP program from the perspective of adolescents impacted by cancer and parents of children or adolescents impacted by cancer, as well as its implementation within a clinical pediatric oncology setting from the perspective of healthcare professionals (HCPs).

## Methods

### Clinical context

The present study was part of a larger, nonrandomized feasibility study conducted at the CHU Ste-Justine (CHUSJ) in Montreal, Canada. The *VIE study* consists of a 2-year, family-oriented HP program that integrates psychosocial support, nutrition education and counselling, and physical activity (PA) interventions. The objective of this program is to optimize health outcomes of children and adolescents impacted by cancer by encouraging adoption of healthy behaviours across the continuum of care. The different interventions’ design is described in details in previous publications (Belanger et al., 2022; Caru et al., 2020b; Ogez et al., 2019) and a summary is provided in Table 1. Children or adolescents newly diagnosed with cancer at the CHUSJ were offered to participate in the program with their family from February 2018 to December 2019 if they were (a) less than 21 years old at diagnosis, (b) treated with radiation therapy or chemotherapy, (c) able to give informed consent (by parents or legal tutors if < 18 years old), and (d) less than 12 weeks postdiagnosis.

**Table 1.** Description of the VIE study program and its components.

Components	Description
Purpose	- The main objective of the VIE project is to demonstrate the relevance and feasibility of an integrated intervention program for children and adolescents newly diagnosed with cancer
	- The project also aims to assess the impact of diet and physical activity on the overall health of children and adolescents with cancer, during and after their treatment trajectory. During the course of the study, the quality of life of the children and their families will be taken into account, equipping them to face the challenges posed by the disease, with the aim of promoting their short- and long-term well-being
Team	- The team includes oncologists, kinesiologists, nutritionists, psychosocial counsellors, physiotherapists, occupational therapists, and social workers
Duration	- VIE study was implemented in the clinical context from February 2018 until December 2021
	- Participants are in the program for 2 years from the moment they are recruited
Interventions *Psychosocial support*	- Aim: To strengthen parents’ sense of control and problem-solving skills training (PSST). Individual sessions focused on PSST, as well as acquiring, developing, and maintaining simple problem-solving skills to meet the needs of families facing childhood cancer. The couple sessions aimed to enhance parents’ communication and resilience by improving their ability to manage real difficulties associated with childhood cancer together
	- Six sessions (i.e. four individual sessions, offered to each parent, and two couple sessions) to support parents of children affected by cancer
	- In blended families, each parent could participate in the program with their new partner and the individual sessions were also offered to single parents
	- A manual for healthcare professionals (provider manual) provided specific instructions to convey the information in a standardized manner. A manual for parents included toolkits for individual and couple sessions, as well as strategies related to communication and dyadic coping
	- This intervention is inspired by problem-solving therapy and has been adapted following the recommendations of the National Cancer Institute. The model and operating hypotheses of this intervention are also derived from previous existing programs and based on cognitive behavioural and systemic theories, developed for this study
	- Provided either at the hospital, at home, or remotely
*Physical activity interventions*	- Aim: To promote physical activity during and after the treatment as well as the adoption of an active lifestyle for the participants and their family
	- Physical activity sessions, goal setting, and counselling for behavioural changes with a team of trained kinesiologists
	- A 2-year follow-up is provided
	- Primary follow-ups every 2 months to allow the implementation of an adapted physical activity program
	- Secondary follow-ups to assess patients’ physical fitness, quality of life, and level of physical activity at four time points (baseline, 1-year post-diagnosis, 2-year post-diagnosis, and end of the study)
	- Six therapeutic education sessions are also offered to patients and their families to provide them with various knowledge and skills related to physical activity. Sessions focus on the following themes: (1) Knowledge of the beneficial effects of exercise on cancer, (2) sport hygiene and recommendations for good practice, (3) sport, physical activity, and sedentary behaviour, (4) physical activity management, (5) physical fitness, and (6) sport autonomy
	- In-person sessions are conducted at the hospital during the medical appointments or hospitalization, or remotely
*Nutritional interventions*	- Aim: To ensure normal growth and development of the patient, weight maintenance after treatment, and prevention of long-term health complications
	- Personalized assessment, goal setting, and counselling with a registered dietitian (RD) focused on addressing nutritional issues related to cancer or treatments and promoting healthy eating behaviours
	- Interventions consist of an initial assessment, follow-up visits planned every 2 months for 1 year, and a 1-year assessment
	- The first meeting with a research RD aimed to establish contact with the participant and his family and create a bond of trust. The initial and 1-year assessments included clinical, anthropometric, biochemical, and nutritional data to describe changes in metabolic health and nutritional status. On follow-up visits, clinical, anthropometric, and nutritional data are collected
	- Throughout the intervention, RDs monitor changes in weight, blood pressure, and diet and provided individualized counselling addressing the patient’s challenges and concerns
	- The intervention approach is based on motivational interviewing, encouraging patients to improve their diet, and parents to accompany their children in their new challenges. The approach also aims at alleviating the food and weight-related guilt of the patients and parents
	- Visits are conducted in the hospital room if the child was hospitalized or at the outpatient oncology clinic. From March 2020 until the end of the project, follow-ups were held by phone due to the COVID-19 pandemic

### Research design

We undertook a qualitative study using semi-structured interviews with adolescents impacted by cancer and parents of children or adolescents impacted by cancer who participated in the HP program, and focus group discussions with HCPs working in the oncology department where the program was implemented.

### Recruitment and sampling

Adolescents and parents in this study were (a) adolescents impacted by cancer ≥14 years old or parents of a child or adolescent impacted by cancer and (b) had been participating in the *VIE study* for at least a year and a half. The first inclusion criteria ensured that participants were old enough to understand and answer interview questions, and the second that they could reflect on their experience in the program during the different treatment phases (i.e. acute, maintenance, and survivorship). We expected to recruit a minimum of 12 families, following a recommendation from Guest, Bunce, and Johnson (Guest et al., 2006), who determined that data saturation generally occurs within the first 12 interviews. Potential participants were contacted by telephone about their willingness to participate in an interview prior to a clinic visit. If they were interested in participating but not available during their visit, a remote interview was proposed. Purposive sampling was used to recruit a variety of participants across different diagnosis groups (leukaemia, lymphoma, and other), age groups (birth-5, 6–12, ≥13 years old), and sex (male, female). The sample was also a convenience sample to the extent that people invited to take part were those attending the clinic when we were doing fieldwork, until reaching data saturation.

HCPs in the present study were clinicians who (a) worked at the CHUSJ during program implementation (i.e. from 2018 to 2021) and (b) had been exposed to the program. Participants were conveniently recruited by email from a list of employees of the oncology department in November 2021. A first round of emails was sent to all potential participants. Then, to have a sample that adequately reflects the diversity of HCPs in pediatric oncology, researchers ensured the sample’s internal diversity by sending another round of emails specifically to members of professions who had not responded in the first round.

### Ethics

Ethical approval for this study was obtained from the CHUSJ Research Ethics Board (#2021-3129, #2021-3278). Written informed consent was obtained from HCPs and parents or adolescents ≥18 years old and verbal consent for adolescents <18 years old. Data were anonymized to ensure confidentiality.

### Data collection

Individual interviews with adolescents and parents were conducted in-person or virtually using the Zoom platform by the first author (CD) from November 2020 to January 2021. A semi-structured interview guideline was developed for this study based on i) literature review, ii) the Theory of Planned Behaviour (TPB) (Ajzen, 1991), and iii) discussion with research team members and a patient partner to ensure its relevance (see Supplemental File). Interviews were conducted in a conversational style, guided by a flexible interview protocol and supplemented by follow-up questions, probes, and comments if needed (DeJonckheere and Vaughn, 2019). Wording of the questions was adjusted based on participants’ age (e.g. adolescents vs parents) to encourage discussion. Themes addressed were (1) factors affecting participation in the program; (2) attitude, subjective norms, perceived behaviour control, and intention to change in relation to the adoption of healthy behaviours; and (3) suggestions of strategies that could support their participation in the program and change in their behaviour. All interviews were audiotaped and transcribed verbatim by the first author (CD).

Focus group discussions with HCPs consisted of semi-structured, open-ended questions on their perspective regarding (1) the program and (2) its implementation (see Supplemental File). Focus groups were audiotaped and transcribed verbatim by the first author (CD). The interview guideline was developed based on the Consolidated Framework for Implementation Research (CFIR) (Damschroder et al., 2009), one of the most recognized frameworks for systematically assessing barriers and facilitators, and identifying improvements to implementation strategies. Discussion was led by one group leader (CD) and one moderator (JK), who also took field notes.

### Data analysis

Descriptive statistics were used to analyse demographic and clinical characteristics of the participants. In terms of qualitative data, thematic analysis was used to capture the perspective of participants*,* which is a method for identifying, analyzing, and reporting patterns (themes) within data (Braun and Clarke, 2006). A basic coding tree was first developed using a deductive approach with the conceptual frameworks (TBP and CFIR). We coded data according to the TBP’s core components (i.e. attitude, subjective norms, behavioural control, intention, and behaviour) and the CFIR’s five domains (intervention characteristics, personal characteristics, inner setting, outer setting, and implementation process). Then, an inductive approach was used to complement the coding tree with general codes constructed from data, using NVivo software (version 1.6.2). The final coding tree was discussed with another author and refined for clarity, and then 20% of data was coded independently by two authors (CD and KL for individual interviews and CD and JK for focus groups). All discrepancies in coding were resolved by discussion and final changes were made to the coding tree. Tobin and Begley principles regarding methodological rigour were used to establish trustworthiness, goodness, and authenticity (Tobin and Begley, 2004), such as member checks, peer debriefing, and keeping an audit trail.

## Findings

### Sample size and participant characteristics

A total of 34 families met the eligibility criteria. Fourteen families were approached, of which two declined participation (no time and no interest), resulting in the inclusion of twelve families (Table 2). As two families had two members who wanted to participate (i.e. one adolescent and one parent), fourteen interviews were conducted. After interviewing 14 participants, saturation was considered reached; participants in the last interviews did not indicate any significant new barriers or facilitators to participation.

**Table 2.** Sociodemographic and clinical characteristics of adolescents and parents.

Characteristics	Adolescents (*n* = 5) *n* (%)	Parents (*n* = 9) *n* (%)
Age of the child at diagnosis (years)		
Birth-4	0 (0)	3 (33)
5–12	0 (0)	4 (44)
13 and +	5 (100)	2 (22)
Sex		
Male	3 (60)	4 (44)
Female	2 (40)	5 (56)
Race/ethnicity		
White/Caucasian	4 (80)	8 (89)
Other	1 (20)	1 (11)
Cancer diagnosis of the child		
Leukaemia	3 (60)	5 (56)
Lymphoma	1 (20)	1 (11)
Central nervous system tumours	0 (0)	0 (0)
Extra cranial solid tumour	1 (20)	3 (33)
Highest level of education attained		
Unfinished high school	-	0 (0)
High school	-	5 (56)
Professional school	-	1 (11)
Entry-level college	-	1 (11)
Graduate school	-	2 (22)
Marital status		
Common-law partners or married	-	8 (89)
Separated	-	1 (11)

Nine (64%) participants were parents and five (36%) were adolescents (mean 15 years old). For seven (58%) families, the child impacted by cancer was a female. Cancer diagnoses represented were leukaemia (*n* = 7; 58%), extracranial solid tumour (*n* = 3; 25%), and lymphoma (*n* = 2; 17%). All children or adolescents were in remission when interviewed and mean time since treatment completion was 11 months. Families were still participating in the program. Interviews ranged in duration between 8 and 38 min (median 13 min).

Eleven HCPs were recruited and two 90-min focus groups were conducted, with five and six participants in each group. All participants were current employees of the oncology department: allied health professionals (*n* = 7, 65%), hospital-based teachers (*n* = 2, 18%), and members of the nursing and medical team (*n* = 2, 18%) (see Table 3). The majority of participants were women (*n* = 8, 72%). All participants had more than 5 years of clinical experience (mean 19 years), including more than 5 years of experience in pediatric oncology (mean 13 years).

**Table 3.** Sociodemographic characteristics of healthcare professionals.

Characteristics	Healthcare professionals (*n* = 11)
Gender – female *n* (%)	8 (73)
Years of clinical experience *mean* (SD)	19 (8)
Years of experience in oncology *mean* (SD)	13 (7)
Discipline *n* (%)	
Nursing and medical teams	2 (18)
Allied health professionals	7 (64)
Hospital-based teachers	2 (18)

### Implementing the program

Three themes were determined when analyzing the data: (1) factors that facilitated participation in and implementation of the program (facilitators), (2) factors that hampered participation in and implementation of the program (barriers), and (3) suggestions provided by participants to improve the program and its implementation (see Table 4 for the coding tree of the first two themes). Quotations supporting the themes will be presented below, using pseudonyms to protect participants’ identity. All quotations were translated from French to English since all interviews were held in French.

**Table 4.** Coding tree for factors affecting participation and implementation.

	Adolescents and parents	Healthcare professionals
**Intervention characteristics**		
Facilitators	- Flexibility and adaptability	- Use of a playful approach
	- Tailored to individuals’ needs	- Enjoyed by participants
	- Perceived benefits of the interventions	- Relevant and answering a need
Barriers	- Perceived difficulty of intervention or burden	- Burden of the intervention for families
	- Too many intervention-deliverers	- Too many intervention-deliverers, not involved for long period of time
		- Perceived lack of training or experience to work with a vulnerable and complex population
**Inner setting**		
Facilitators	- Convenience	- Cultural shift towards health promotion
	- Support from HCPs	- Complementarity of some interventions with clinical care
Barriers	- Time or organizational constraints	- Added burden to clinician’s work
	- Lack of integration in clinical care	- Negative implementation climate
		- Lack of collaboration and interdisciplinary work between teams
		- Difficulty to embed intervention in clinical care
		- Overlap of some interventions
**Outer setting**		
Facilitators	- COVID-19 pandemic led to increase the offer for online interventions, which was appreciated by some participants	- Gap in health promotion interventions or services in this population
		- COVID-19 pandemic led to an increased use of technology and at-home interventions
Barriers	- Demotivation due to the pandemic or no interest in online interventions	- COVID-19 pandemic hampered communication and collaboration between teams
**Characteristics of individuals**		
Facilitators	- Knowledge and positive beliefs about intervention	- Trusting relationship established with individuals involved in the long term
	- Social support	
	- Trusting relationships with intervention-deliverers	
Barriers	- Health issues (physical and mental)	- Lack of need for these interventions as perceived by families, possible lack of knowledge
	- Lack of need	
	- Lack of interest or motivation	
**Implementation process**		
Facilitators		- Identification of some clinicians as project champions before the implementation
Barriers	- Inconsistency in the delivery of interventions across time	- Lack of publicity for some interventions
		- Lack of knowledge of clinicians regarding the interventions and the study in general (e.g. goals, criteria, and interventions)
		- Lack of engagement and involvement of clinicians in the different steps (i.e. intervention development, planning, implementation, and evaluation)

### Facilitators to participation and implementation

Some elements of the inner setting (i.e. oncology department) were viewed as facilitators. Families had long waiting hours both at the clinic and during their hospitalization, and they were happy to fill those hours with HP interventions.

‘We're here, we’re available, that fills up the days too, because it helps him get through the waiting times […] it changes the day’ (Steve, parent of a 4-year-old).

Characteristics of the interventions that were reported as facilitators were the flexibility and adaptability in the delivery of the program, and the tailoring of interventions to their individuals’ needs. Interventions could also be delivered remotely (i.e. Zoom meetings or phone), which was viewed as very convenient, especially considering the Coronavirus disease 2019 (COVID-19) pandemic that occurred during the second half of the program. Regarding personal characteristics, having a good understanding of potential benefits of the interventions or a positive attitude towards the adoption of healthy behaviours motivated participation in the program and improved the overall experience of its advantages.

‘Of course, it helped me to sustain healthy behaviours. Doing some exercises, once in a while, things like that, because if I had only listened to myself…’ (Kevin, 16-year-old participant).

‘I have seen the importance of being active... we will keep this new way of living’ (Kim, parent of a 5-year-old).

Families reported that the opinion of significant others, or the subjective norms according to the TPB, influenced their participation. Having positive relationships and trust in the research team members was a motivation for participating as well as including siblings or other family members in the interventions. When questioned on opinion leaders, defined in the CFIR as individuals in an organization who influence attitudes and beliefs towards an intervention, most parents or adolescents said they had received support from members of the clinical team to participate.

‘I would say they were all in favor of [the VIE study]. When you talk to the nurses and all, they talk about it with interest… they are enthusiastic about this program…’ (Catherine, parent of a 13-year-old.^1^)

HCPs perceived that there was a need for this type of intervention in the clinical setting. They felt that the type of interventions was well-chosen and that they were filling a clinical gap, especially towards the end of treatment phase or during survivorship, when clinicians are less involved with their patients.

‘When they presented the project to us, I found that it looked relevant for the families, to be able to empower them in various aspects of their lives in which it was difficult to keep a balance’ (Emilie, allied health professional).

An HCP also mentioned that implementing those interventions supported a much needed cultural shift towards the promotion of healthy behaviours in the oncology department, not just a focus on recovery and medical aspects.

‘When I started working here, what was important was that children eat during chemotherapy treatments. They could eat anything, but they had to eat. So, the VIE study for me, it was like “wow.” We can tell people, you can eat well, we are going to give you the best advice possible, so you can eat better and better’ (Sarah, allied health professional).

Another aspect that encouraged HCPs to support the interventions was the fact that the patients appreciated and benefited from the interventions. The playful aspect of the PA interventions was particularly appreciated by younger participants and helped adolescents get back to ‘normal life’.

‘From the comments that I had from patients, it was extra appreciated’ (Adam, member of the nursing and medical team).

Overall, facilitators to participation in and implementation of the program related to the inner setting, characteristics of the interventions, and characteristics of individuals (i.e. themselves).

### Barriers to participation and implementation

The most important barriers to participation reported by families were time and organizational constraints, which were related to the inner setting of the program. Other barriers related mostly to their individual characteristics, including lack of interest or need, physical or mental health issues, and perceived difficulty of the intervention or burden. For some parents, feeling overwhelmed hampered their participation, particularly at the beginning of the cancer journey when treatments are intensive and the shock of the cancer diagnosis still present.

‘You know, of course it is very challenging, in a moment where… it is not going really well… when you know you have already lost balance and you are trying to get back on your feet and it is difficult… and you have homework given to you […] you are already psychologically exhausted and you have to do that on top of it…’ (Myriam, parent of a 5-year-old).

Age of the children or adolescents impacted by cancer was also noted as an important factor impacting their level of participation and motivation. Barriers related to personal characteristics, particularly lack of motivation, were reported more frequently by adolescents or parents of adolescents compared to parents of younger children.

‘If he had not been an old teenager, also […], you know if he had been younger, like 10 or 12 years old, he probably would have felt the need to be active’ (Nadia, parent of a 16-year-old).

HCPs have also identified challenges and barriers during the implementation process. Barriers that were identified by HCPs were the lack of publicity for certain elements (e.g. nutrition workshops) and the fact that the HCPs in general had a low level of knowledge of what the interventions entailed. This prevented them from acting as facilitators or from championing the interventions with their patients. HCPs felt that their high-level of expertise in the field had not been considered when developing and implementing the interventions. This resulted in them not feeling involved in the project and overlooked.

‘We all recognize the relevance […], but it is how to implement it so that it can be beneficial for everybody and realistic in the clinical context, which is the most important’ (Simon, member of the nursing and medical team).

Regarding the intervention characteristics, the main barriers identified by HCPs were the added burden both for them and the families and negative impact on their work. HCPs stressed the difficultly of embedding the research interventions in the clinical context:

‘It was a great project, enjoyed by the patients. But it was extremely demanding from a clinician’s point of view’ (Adam, member of the nursing and medical team).

‘Regarding my work, it was conflictual […], I was sometimes not able to do my work’ (Nadine, allied health professional).

Some interventions were perceived as overlapping and competing, rather than complementing each other. HCPs mentioned that it has been difficult to establish a trusting relationship with some research team members, given the vast variety of different interventions that were delivered and that some intervention deliverers (e.g. trainees) had little experience or training in the field. The lack of coordination, collaboration, and communication between the research and clinical teams was viewed as an important barrier. One parent also mentioned the difficulty navigating all the professionals involved in the study, which complicates the establishment of meaningful and trusting relationships with everyone.

‘There are already so many stakeholders who gravitate that at some point, making effort everywhere, we become exhausted and hum… we struggle to see the added value of everybody’ (Paul, parent of a 13-year-old).

Overall, barriers to participation in the program related mainly to the inner setting and individual characteristics, whereas barriers to implementation related to the implementation process and characteristics of the interventions.

### Suggestions for improvement

Participants suggested various ways of improving the program. The most common suggestion from both families and HCPs was to better integrate HP interventions into clinical care. A parent even suggested making HP interventions mandatory.

‘If it is that important to adopt healthy behaviours, well… it should not be done on a voluntary basis. It has to be part of the cancer treatment […] more imposed than optional’ (Joanne, parent of a 4-year-old).

HCPs want to be involved in the different project phases, which necessitate a clear definition of each professional’s role before implementation. When asked about how she felt regarding a future implementation of the project in her clinical context, an HCP said:

‘If it is exactly the same… I am not really enthusiastic. If the roles are well-defined, everybody’s tasks, the why, the how, the timing [then, yes]’ (Christine, allied health professional).

The lack of clear information was also felt by participating adolescents and families. One adolescent suggested that being informed of the clear purpose and goals of the interventions could increase participants’ motivation.

‘I think it could have helped me to understand why I was doing this, not just “you have to do it”’ (Noah, 18-year-old participant).

Another idea proposed by an HCP would be to develop group activities that focus on educating children and their families regarding the adoption of healthy nutritional and PA behaviours.

‘Maybe there is another way, other ways that it could be implemented […] If you had […] a small group activity that we could develop […] on a day that we have a number of the same age group, we could find [… ] a workshop that will talk about nutrition, to the kid, and with his parent’ (Sophie, allied health professional).

HCPs suggested revising the admissibility criteria and adapting the interventions for each type of diagnosis, treatment regimen, or age. Timing of intervention delivery could also happen later in the cancer continuum, for patients at the end of the cancer continuum.

‘But I think that from my clinical experience, hum, in the continuum of care […], I would see this coming a little bit later. To let the ‘storm’ of the diagnosis pass, to pass the acute adverse effects, then can we maintain the healthy behaviours?’ (Anna, allied health professional).

Suggestions for improvements mainly related to improving the implementation process and better adapting some of the proposed interventions’ characteristics to the local context.

## Discussion

This study allowed us to identify the factors affecting participation in an HP program and its implementation in a clinical context, from the perspectives of participants in the program and HCPs. By employing a qualitative approach, we gained insights into how families navigated participating in the program in addition to pediatric cancer treatment while also highlighting critical factors that can enhance or impede the integration of HP interventions into routine clinical care. Our results highlight the need for HP interventions but also the challenge of successfully embedding such innovative interventions into a highly medical clinical setting.

Participants reported several facilitators that supported their engagement in the HP program, including the social context and enjoyment of activities such as playing games with siblings during physical activity interventions. Interpersonal relationships played a positive role in fostering healthy behaviours. This finding aligns with previous literature demonstrating that enjoyment during exercise was the main motivation for children with cancer during treatment (Gotte et al., 2014) and that social interactions were important to the overall PA experience (Craike et al., 2013). Families appreciated the program’s adaptability and how individualized interventions were tailored to the specific needs of children and families. Our findings foreground the crucial importance of adaptability of HP program in overcoming some of the barriers to behaviour change reported in other studies, such as fatigue, organizational constraints, or negative feelings about one’s self (Ross et al., 2018).

Similar to our results, another exercise-based rehabilitation program within an adult cancer unit was perceived by clinicians to initiate a cultural change (Dennett et al., 2021). In this study, key practical elements for success included delivering consistent, positive messaging about exercise from a broad range of hospital staff and facilitated by the fact that it was filling a service gap. To generate a sustainable culture shift, all of those involved (including HCPs and participants) should be well-informed about the interventions and involved in their development and/or implementation. HCPs reported challenges related to their lack of knowledge about the HP interventions, which limited their ability to advocate for these interventions effectively. This disconnect underscores the need for comprehensive training and clear communication between research teams and clinical staff to enhance understanding and support for HP initiatives. Not having a good understanding of the interventions and their aim also interfered with participants’ engagement.

Families expressed that time constraints and cancer treatment’s burden often overwhelmed them, leading to reduced engagement with HP interventions. This population is confronted with complex emotional and organizational challenges (Beaulieu-Gagnon et al., 2019a). Those barriers were heightened at the beginning of the cancer care continuum, a time when families were often disrupted and consumed by the intensity of cancer therapy (Ross et al., 2018; Van Dijk-Lokkart et al., 2015). This suggests that the timing of interventions needs to be more strategic within the cancer care continuum as well as flexible. A practical solution offered by HCPs was to revisit the onset of some interventions, such as a staggered approach in the implementation of the different interventions given the needs of the families or even lessening their intensity during the acute phase of treatment. Focusing on the survivorship phase, where patients often experience a service gap was also identified as a suitable alternative, especially in the case of educational activities on the long-term benefits of adopting healthy behaviours.

Another difficulty faced by the research team was that adolescents in our study showed less interest or motivation toward behaviour change interventions. Similarly, another study investigating attitudes towards improving lifestyle behaviour after cancer treatment found that parents were more interested in having their children participate than adolescents themselves (Touyz et al., 2019). Thus, developing targeted strategies that are age-specific for adolescents or providing additional support will be crucial in future initiatives to fully engage them in such interventions.

Adding educational activities such as group workshops on specific subjects was suggested by participants and could be one way to increase their intention to change. According to Götte et al. (2014), educational strategies can be used to inform children impacted by cancer and their families about how barriers such as lack of energy and bad mood can be influenced by PA and thus improve their participation in the program. Our results also suggest that education should go beyond the sole dissemination of information to also include the fostering of participants’ meaning-making and of taking an active role in their behaviour change. It is important to note that in-person nutrition education and cooking workshops have been developed and tested in our clinical context but remain difficult to implement (Beaulieu-Gagnon et al., 2019b).

When deciding on the timing of interventions, meeting participants where they are at in the change process is important, and the use of theory-based behaviour change strategies could support this (Brown et al., 2020). In this way, participants’ actual readiness to change can be addressed instead of addressing generic change in health behaviours prematurely and ineffectively. Interventions that act by changing attitudes towards health behaviours may have a lasting impact on intention to change or engagement (Hullmann et al., 2021). For example, providing extensive training in motivational interviewing (Dewez et al., 2021; Miller and Rollnick, 1991) to intervention providers or integrating Prochaska’s stages of change in their practice could be helpful. A study using a motivational intervention in childhood cancer survivors found a significant improvement in PA and secondary outcomes such as cancer-related fatigue and quality of life (Cheung et al., 2022).

### Study limitations

As a single-centre study, findings cannot be generalized to the entire pediatric cancer population and HCPs, which may limit the transferability of the recommendations. Despite a small sample size, we aimed to provide a range of perspectives on the participation in and implementation of the program to foster transferability of results into other contexts. There may be some degree of selection bias in our sample, that is, individuals who agreed to be interviewed may differ in important ways from those who did not or from individuals who dropped out of the study. Another limitation is the fact that the first author, who conducted and analysed the data, was a member of the research team, which could have introduced bias. The interviewer was aware of her biases and kept them in mind when collecting and analyzing data. For the interviews, this may have limited participants’ willingness to share negative experiences due to the social desirability. We attempted to avoid the personal bias in the data analysis by triangulating the data. The second person who analysed the data was not part of the research team and had limited knowledge of the clinical context. Finally, in this study, the program was evaluated as a whole, making it challenging to distinguish between its various components.

### Implications for practice

Participants provided valuable suggestions for improving the integration of HP interventions into standard care. Both families and HCPs advocated for a more structured approach and the relevance of the mandatory participation in HP programs as part of treatment plans. This perspective highlights a shift in thinking towards recognizing health promotion as an essential component of cancer care rather than being overlooked as an optional add-on, filling in the waiting time of the families impacted by cancer. Additionally, better defining the roles and responsibilities of HCPs within the intervention framework emerged as a critical need for facilitating successful implementation. Finally, working on changing families’ and HCPs’ perception regarding the importance and necessity of integrating HP interventions in clinical care to optimize health outcomes is crucial. The next steps for our team will involve revising the program based on the feedback received and implementing it again, while evaluating its effectiveness using a more rigorous study design. In the long term, we plan for the HP interventions to be sustainably integrated into clinical practice.

## Conclusions

This study provides researchers and clinicians several insights into the perspective of adolescents impacted by cancer, parents of children or adolescents impacted by cancer, and healthcare professionals (HCPs) to participate in and implement effectively health promotion interventions in clinical care. By addressing barriers and leveraging facilitators, we can create a supportive environment that fosters healthier behaviours throughout the cancer continue and contribute to improving health services and quality of life for childhood cancer survivors.

## Supplemental Material

Supplemental Material - Implementing health promotion interventions in a pediatric oncology setting: A qualitative study among families impacted by cancer and healthcare professionals Supplemental Material for Implementing health promotion interventions in a pediatric oncology setting: A qualitative study among families impacted by cancer and healthcare professionals by Catherine Demers, Isabelle Gélinas, Johanne Kerba, Keven Lee, Claude Julie Bourque, Kristopher Lamore, Isabelle Bouchard, Caroline Meloche, Caroline Laverdière, Daniel Curnier, Valérie Marcil, Serge Sultan, Daniel Sinnett, and Johanne Higgins in Journal of Child Health Care

## Data Availability

The datasets used and/or analysed during the current study are available from the corresponding author on reasonable request.*

## Note

1. Age at diagnosis.
