# Supplementary material for: Implementing health promotion interventions in a pediatric oncology setting: A qualitative study among families impacted by cancer and healthcare professionals
Source: J Child Health Care. 2025 May 8;30(2):260–77. doi: 10.1177/13674935251341008 (PMC13168591; doi:10.1177/13674935251341008)
Supplement: Supplemental Material - Implementing health promotion interventions in a pediatric oncology setting: A qualitative study among families impacted by cancer and healthcare professionals [file sj-pdf-1-chc-10.1177_13674935251341008.pdf]

Supplementary File

Interview guides for semi-structured interviews and focus group discussions

Individual interviews

Introduction: You've been part of the VIE Project for about a year now, and we'd like to get a better idea of how you're experiencing it. So I'd like to take a moment with you to talk about it. The length of the interview will depend on the exchanges we have, but on average it lasts about 30 minutes.

1. Why did you agree to take part in the VIE project?
2. Can you tell me about your experience with the VIE project so far?
3. What motivates you to participate in the various components? Or on the contrary, what might limit your participation?
4. What is the reaction of those around you or of the medical team as a whole to your participation in this project (e.g. encouragement, comments, reluctance, etc.)?

As you know, the aim of the VIE project is to change the way children with cancer and their families are cared for, in order to reduce the negative effects of the disease and treatment during and after the care episode.

5. How would you describe the importance of healthy lifestyle habits for you?
6. What do you think the VIE project has brought you so far?
7. In the long term (e.g. after treatment), what do you think the VIE project will bring you?
8. What challenges or irritants have you encountered during your participation in the project?

9. As you know, the VIE project is a research project. What advice or suggestions could you give us to help us improve the project (e.g. how to approach you, frequency of interventions, etc.)?

Focus group discussions

1. What did you think of the VIE project as a whole?

- Prompts: What did you find positive? What were the limitations?

2. What changes or modifications could be made to the program to improve it?

- Prompts: Would it be feasible, in your opinion, to make these modifications?

3. Are there any aspects that you feel should not be modified?

- Prompts: Which ones and why?

4. To what extent do you think the interventions proposed by the VIE project met a need?

- Prompts: Needs of patients or caregivers? How did it meet their needs?

5. To what extent do you think the VIE project had an impact on your patients?

- Prompt: At what level (e.g. lifestyle, health, etc.)?

6. How receptive have Charles Bruneau staff been to the implementation of VIE project interventions?

- Prompt: Why? Has receptiveness changed over time? Which units were the most appropriate for implementation (e.g. outpatient clinic vs. floors)?

7. To what extent do you find the VIE project's interventions compatible or “fit” with current practices and operations in the clinical environment?

- Prompts: What complications could arise? What impact would this have on your work?

8. How do you feel about these interventions being implemented in your clinical environment in the future?

- Prompts: Do you anticipate? Are you enthusiastic? What reasons would prompt you to recommend, or not, implementing this project at Ste-Justine? At another facility?

9. In your opinion, who should be involved in a successful implementation?

- Prompts: Why? How could they help?
